# Cyclic Damage Accumulation in the Femoral Constructs Made With Cephalomedullary Nails

**DOI:** 10.3389/fbioe.2020.593609

**Published:** 2021-02-05

**Authors:** Farah Hamandi, Alyssa Whitney, Mark H. Stouffer, Michael J. Prayson, Jörn Rittweger, Tarun Goswami

**Affiliations:** ^1^Department of Biomedical, Industrial, and Human Factors Engineering, Wright State University, Dayton, OH, United States; ^2^Department of Orthopaedic Surgery, Sports Medicine and Rehabilitation, Wright State University, Dayton, OH, United States; ^3^German Aerospace Center, Institute of Aerospace Medicine, Cologne, Germany

**Keywords:** femur, cephalomedullary nail, damage accumulation, biomechanics, gender

## Abstract

**Background:** The purpose of this study was to evaluate the risk of peri-prosthetic fracture of constructs made with cephalomedullary (CM) long and short nails. The nails were made with titanium alloy (Ti-6Al-4V) and stainless steel (SS 316L).

**Methods:** Biomechanical evaluation of CM nail constructs was carried out with regard to post-primary healing to determine the risk of peri-implant/peri-prosthetic fractures. Therefore, this research comprised of, non-fractured, twenty-eight pairs of cadaveric femora that were randomized and implanted with four types of fixation CM nails resulting in four groups. These constructs were cyclically tested in bi-axial mode for up to 30,000 cycles. All the samples were then loaded to failure to measure failure loads. Three frameworks were carried out through this investigation, Michaelis–Menten, phenomenological, and probabilistic Monte Carlo simulation to model and predict damage accumulation.

**Findings:** Damage accumulation resulting from bi-axial cyclic loading in terms of construct stiffness was represented by Michaelis–Menten equation, and the statistical analysis demonstrated that one model can explain the damage accumulation during cyclic load for all four groups of constructs (*P* > 0.05). A two-stage stiffness drop was observed. The short stainless steel had a significantly higher average damage (0.94) than the short titanium nails (0.90, *P* < 0.05). Long titanium nail group did not differ substantially from the short stainless steel nails (*P* > 0.05). Results showed gender had a significant effect on load to failure in both torsional and bending tests (*P* < 0.05 and *P* < 0.001, respectively).

**Interpretation:** Kaplan–Meier survival analysis supports the use of short titanium CM nail. We recommend that clinical decisions should take age and gender into consideration in the selection of implants.

## Introduction

Nearly 30% of the population between the ages of 70–75 years experiences a femoral fracture (Johnell and Kanis, 2006). This rate is projected to double by 2040 as population in this age group increases (Centers for Disease Control Prevention, 2018). Approximately 65 million patients in the United States age 60 and older suffer from bone diseases that lead to fractures (Royal College of Physicians, 2017). The CM nailing is standard treatment for surgical stabilization of proximal femur fractures, using either a long or a short CM nail depending on where the fracture occurs subtrochanteric or intertrochanteric, respectively. The CM nails have different shapes, sizes, number of screws needed to lock the construct, and insertion methods specified by manufacturers. In the late 80s, the short nail was introduced, then a long nail that ends at the supracondylar region was introduced (Bong et al., 2006).

The CM nail has been featured in literature describing the clinical outcomes for long (Kuzyk et al., 2012; Kane et al., 2014; Vopat et al., 2015; Jin et al., 2018; Mukherjee et al., 2019) and short (Jones et al., 2006; El-Dessokey and Mohammed, 2013; Kwak et al., 2018) nails in general (Hou et al., 2013; Okcu et al., 2013; Boone et al., 2014; Kleweno et al., 2014; Vaughn et al., 2015; Dunn et al., 2016; Horwitz et al., 2016). Okcu et al. (2013) compared between 15 short nails versus 18 long nails and indicated that the time of the operation was longer in the fixation with long nails, though they needed shorter follow up. Kleweno et al. (2014) performed a retrospective study on 219 short nails versus 340 long nails and demonstrated that failure rates were similar for both nails in older patients (>65). Another retrospective study performed by Hou et al. (2013) on 183 long and 100 short nails indicated that long nails needed more operation time with high blood loss while a delayed union was observed on short nails. Additionally, they concluded that the benefits were similar for short and long nails in older patients. Boone et al. (2014) performed a retrospective study on 82 short and 119 long nails and indicated that long nails operations needed more time while there were no other significant differences. Vaughn et al. (2015) demonstrated that short nails had significantly higher risks of failure than long nails. In general, short nails pros represented by simple insertion while thigh pain and distal fracture are the main cons. On the other hand, the mechanical benefits are the main pros of long nails while long operation time and high radiation exposure are the main cons.

From a biomechanical perspective, investigating short and long nails following pertrochanteric fracture consolidation was reported by Breceda et al. (2020) and concluded that the nail design does not have significant effects for high age groups (83.4 ± 7.7 years old). Nevertheless, it is important to point that their work neglects the effect of the demographical factors. Additionally, Marmor et al. (2015) investigated the short and long nails where their work was based on synthetic bone. In their study, they tested synthetic bone for ten cycles at 0.2 Hz and loaded to failure, and they reported the results only in axial condition. The cyclic behavior of the bone and the bone-nail construct depends on how the load was applied, uniaxial versus biaxial (Schneider et al., 2001). After the surgery, the bone-construct experiences a combined compression and torsion type of loading (Schneider et al., 2001). Therefore, it is important to design experiments that simulate conditions experienced *in vivo*. Since the stiffness has been related in the literature to have an effect on the life of the construct (Bottlang et al., 2010; Schmidt et al., 2013; Nanavati and Walker, 2014), we used stiffness as a controlling parameter in assessing the construct behavior. Since fracture union likely takes place in approximately 14–15 weeks, and from 6th week post-surgery rehab exercises allow partial to full load-bear and walking, allowing the construct to accrue damage cyclically. Literature has incidence of premature device failures post-surgery by peri-implant or peri-prosthetic fractures during the 15 weeks (Lewallen and Peterson, 1985; Jain et al., 2004; Nisar et al., 2013), there is a need to understand how damage accrues with number of cycles in two cyclic life times investigated in this paper using in-tact constructs assumed to have been healed.

The objective of this study was to perform biomechanical evaluation of CM nailed constructs under biaxial cyclic loading to demonstrate the integrity of non-fractured femora simulating post-primary healing for an additional life cycle, for 30,000 cycles. Subsequently, demonstrate that the axial load to failure of constructed femora ranging from 5 to 10 of cyclic conditions depending upon the bone quality and gender. Flexural fatigue and static strength were determined, and the same conditions proven as that of biaxial. The biomechanical evaluation of the two types of constructs (pre/post healing) with short or long nails have not reported in literature discussing the peri-implant and/or peri-prosthetic fractures and resulting construct efficacy. An attempt was made to mathematically predict the cyclic damage development using the stiffness parameter for these constructs. Such models do not exist in the literature that will be very beneficial in predicting and/or developing new experimental programs. This research motivated by that gap in knowledge yet to apply the results obtained in this experimental program in surgery. Damage development in the constructs was modeled in terms of stiffness, and prediction models developed. Since in pre-primary healing, fixation efficacy, and joint stability is demonstrated by how much the joint gap decreases over time or cycles, stiffness drop during cycling proposed to describe the process. The risk of peri-prosthetic fracture of long versus short nails using titanium and stainless-steel materials, in four groups, was determined from failure data for each gender probabilistically.

## Materials and Methods

### Cadaver Bones Preparation

**FIGURE 1:**
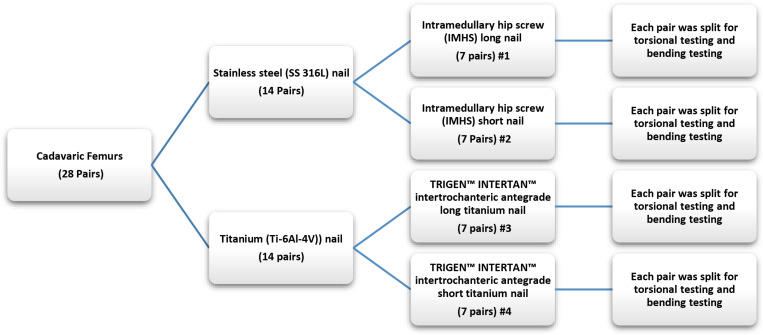
Organization and test plan for each pair of femurs.

The experimental work was performed at the biomechanics laboratory located at Miami Valley Hospital in Dayton, OH, United States to evaluate the risk of peri-prosthetic fracture of non-fractured femora constructs. Similar procedure was previously performed on synthetic femurs to investigate different types of fixation (Goswami et al., 2011) using various osteotomy gaps and hybrid-fixation. Biomechanical evaluation of CM nail constructs was carried out with regard to post-primary healing to determine the risk of peri-implant/peri-prosthetic fractures. In this study, twenty-eight pairs of cadaveric femurs were harvested. Each pair of femurs was randomized to receive a fixation with either an intramedullary hip screw (IMHS) short stainless steel nail (seven pairs), intramedullary hip screw long stainless steel nail (seven pairs), TRIGEN INTERTAN intertrochanteric antegrade short titanium nail (seven pairs) or TRIGEN INTERTAN intertrochanteric antegrade long titanium nail (seven pairs), all manufactured by Smith and Nephew, Memphis, TN, United States. Then each pair was split for axial-torsional and bending cyclic testing. The details of the devices are displayed in Table 1 and shown in Figure 1.

**TABLE 1:** Details of the intramedullary hip screw nails used in the experiment and group numbers (#).

Device length	Material	Company	No. of devices
Long #1	Stainless steel (SS 316L)	Smith and Nephew	7 Pairs
Short #2	Stainless steel (SS 316L)	Smith and Nephew	7 Pairs
Long #3	Titanium alloy (Ti-6Al-4V)	Smith and Nephew	7 Pairs
Short #4	Titanium alloy (Ti-6Al-4V)	Smith and Nephew	7 Pairs

### Biomechanical Testing Procedure

**FIGURE 2:**
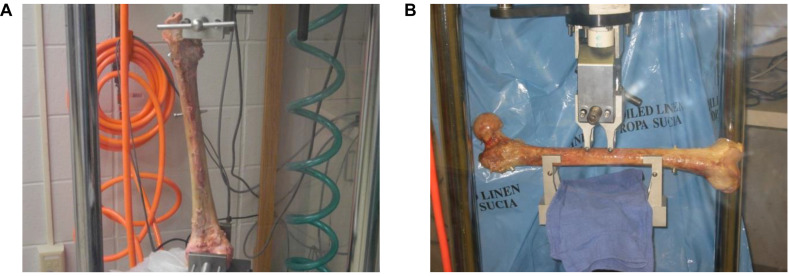
**(A)** Testing set-up for a single-leg stance with torque applied at the distal end of the femur, **(B)** Testing set-up for four-point bending moment along the femur shaft (Under ASTM D6272-17e 1 under *B* testing type).

The testing was performed by an EnduraTEC servopneumatic machine (ElectroForce Systems Group, Bose Corporation, Eden Prairie, MN, United States). One side of each pair was tested in simultaneous axial compression at 700N to simulate single-leg stance, where torque produced during walking was simulated by applying ±5 Nm (Taylor and Walker, 2001) for 30,000 cycles at 2 Hz. Axial compression was applied at the femoral head while torque was applied at the distal end, both attaining peak values at the same time. At the end of cyclic fatigue tests, the femur was gripped at the head and the distal end and loaded to failure (Figure 2). The matched pair was tested in four-point bending at 830N to simulate the moment produced during walking (Taylor and Walker, 2001) for 30,000 cycles at 2 Hz, and then loaded to failure by the same mechanism following ASTM D6272-17e 1 under *B* testing type. This testing was performed by applying the load to the center of the femoral shaft on the medial side (Figure 2). Stiffness, energy change, and load-to-failure were analyzed to identify any difference between the four groups.

The failure criterion in this research is multi-fold:

1. Under dynamic conditions, cyclic axial-torsional fatigue, the 30,000 cycles would best describe an additional lifetime of 14 weeks of recovery. At the end of this time the construct is assumed to have been healed, no longer susceptible to failure.
2. Under static condition, the load to failure was carried out, upon the construct had been pretested in fatigue. Thus, pre-fatigue constructs, all underwent 30,000 cycles, were tested to failure and the load was determined. Such tests also enable the nature of the failure via oblique cracks running perpendicular to bending load application and femoral neck fractures. Therefore, these observations were directed to support the clinical usage in the operation room by recommending the efficacy of one device over the other based on the stiffness drop.
3. Torque and flexural load to failure determined to simulate the rotation of the distal femur and bending of mid-shaft failure so that those critical parameters avoided during the rehabilitation.
4. Stiffness drop 80% at the end of 1000 cycles was assumed to define the failure. Since the stiffness provides the construct stability and fixation outcome, where a healing had taken place, this parameter was used to control the 28 weeks of recovery.

### Statistical Analysis

The statistical analysis was performed with JMP14 (SAS Institute Inc., NC, United States) and MATLAB R2019a (Natick, MA, United States: The MathWorks Inc.) programs. The stiffness, load to failure, number of cycles to failure, and damage accumulation were documented for each construct and checked for normality. One-way analysis of variance (ANOVA) with a statistical significance set at a *p*-value < 0.05 was performed. Paired Student’s *t*-test was used to evaluate the significant differences between the four groups of CM nail fixation with respect to age, gender, length, and material properties. Kaplan–Meier analyses were performed to predict construct survival significant difference between the four groups.

### Damage Accumulation

Cumulative cyclic damage accumulation with respect to the number of cycles is very useful analysis to predict the construct efficiency. This data may be used to design rehabilitation schedules and restrict load bear. Such models do not exist in the literature as most efforts are directed by applications in surgery. We used various empirical approaches to predict composite damage for four constructs using the following:

#### Michaelis–Menten Model

In order to achieve clinical relevance in the biomechanical testing of a bone and implant construct, current research protocols utilize high numbers of cycles to simulate months of use after a device has been implanted. For instance, average number of steps walked during a typical year is assumed to be one million. Therefore, assuming 20,000 steps per week will translate to 80,000 fatigue cycles per month for the femur construct. The majority of femur studies fall somewhere within this range of fatigue cycles. In order to help choosing an appropriate number of cycles to attain clinical relevance, it is important to understand the fatigue mechanism the bone/construct should resist.

In order to analyze the damage accumulation, damage (D) was defined as:

(1) $$D=1-\left(\frac{\xi_{T}}{\xi_{O\,T}}\right)$$

Where **ξ**_*T*_ is the instantaneous total stiffness at a given cycle and **ξ**_*OT*_ is the total stiffness under bi-axial, and flexural fatigue conditions.

Equation 1 shows that the damage is a function of the stiffness ratio, prior to cycling it is 0, and as the cycles increase it increases and assumed to reach 1 at failure. Therefore, stiffness is very important to pre-healing and triggering union/non-union in the surgeries. If we control the stiffness, the fracture fixation and the stability of the fixation device will be ensured.

During the initial load bear, the construct undergoes stiffness changes, where early cyclic action results in higher stiffness drop. When the stiffness behavior is modeled with respect to fatigue cycles; we observe a two stage behavior, where stage I represents the stiffness reduction stage and stage II represents stiffness steady state behavior. Therefore, the two stage stiffness drop, presents a potential parameter in which to represent damage, that accrues at different rates.

#### Damage Accumulation – Phenomenological Model

A Regression model was developed for bone damage accumulation with respect to stiffness and bone mineral density using MATLAB. The regression model is,

(2) $$D={\text{a}}_{0}-\left({\text{a}}_{1}\,\,\xi_{A}\right)+\left({\text{a}}_{2}\,\rho \right)-\left({\text{a}}_{3}\,\,\xi_{A}^{2}\right)+\left({\text{a}}_{4}\,\,\xi_{A}\,\rho \right)-\left({\text{a}}_{5}\,\rho^{2}\right)+\left({\text{a}}_{6}\,\,\xi_{A}^{3}\right)-\left({\text{a}}_{7}\,\xi_{A}^{2}\,\rho \right)-\left({\text{a}}_{8}\,\,\xi_{A}\,\rho^{2}\right)$$

Where *D* denotes the damage accumulation of the bone, **ξ**_*A*_ is the axial stiffness, ρ is the bone mineral density, *a*_0_,*a*_1_,*a*_2_,*a*_3_,*a*_4_,*a*_5_,*a*_6_,*a*_7_, and*a*_8_ are empirical coefficients. A comparison between the prediction profiler of damage accumulation with respect to load to failure in both bending and tension tests was performed and found to be a function of stiffness parameters and density.

A comparison between the prediction profiler of damage accumulation with respect to load to failure in both bending and torsion was performed and found to be a function of stiffness parameters and density. The prediction expression of damage accumulation with respect to load to failure in bending is as follows:

(3) $$D={\text{b}}_{0}+b_{1}\,\xi_{A\,O}+{\text{b}}_{2}\,\xi_{A}+{\text{b}}_{3}\times \left({\text{ξ}}_{A\,O}-{\text{b}}_{4}\right)\,\times \,\left({\text{ξ}}_{A}-{\text{b}}_{5}\right)+b_{6}\times {ℒ}_{B}+{\text{b}}_{7}\times \,\left({\text{ξ}}_{A}-{\text{b}}_{8}\right)\times \left({ℒ}_{B}-{\text{b}}_{9}\right)+b_{10}\,\rho +{\text{b}}_{11}\times {\left({ℒ}_{B}-{\text{b}}_{12}\right)}^{*}\,\,\left(\rho -{\text{b}}_{13}\right)$$

Where _ξ*A*_ is the instantaneous axial stiffness, **ξ**_*OA*_ is the original axial stiffness, ρ is the bone mineral density, _*ℒB*_ is load to failure in bending, *b*_0_,*b*_2_,*b*_3_,*b*_4_,*b*_5_,*b*_6_,*b*_7_,*b*_8_,*b*_9_,*b*_10_,*b*_11_,*b*_12_, and*b*_13_ are empirical coefficients. Additionally, the prediction expression of damage accumulation with respect to load to failure in torsion is as follows:

(4) $$D=c_{0}{\text{ξ}}_{A\,O}+c_{1}{\text{ξ}}_{A}+c_{2}{ℒ}_{T}+c_{3}{\left({\text{ξ}}_{A\,O}-c_{4}\right)}^{*}({ℒ}_{T}-c_{5}+c_{6}{\left({\text{ξ}}_{A\,O}-c_{7}\right)}^{*}\,\,\left({ℒ}_{T}-c_{8}\right)+c_{9}\,\rho +\,c_{10}\,\,{\left({ℒ}_{T}-c_{11}\right)}^{*}\,\,\left(\rho -c_{12}\right)\,$$

Where **ξ**_*A*_ is the instantaneous axial stiffness, _ξ *AO*_ is the original axial stiffness, ρ is the bone mineral density, _*ℒT*_ is load to failure in torsion, and *c*_0_, *c*_1_, *c*_2_, *c*_3_, *c*_4_, *c*_5_, *c*_6_, *c*_7_, *c*_8_, *c*_9_, *c*_10_,*c*_11_,*andc*_12_ are empirical coefficients.

#### Probabilistic Damage Accumulation – Using Monte Carlo Simulation

The Monte Carlo simulations were performed in JMP14 to produce a set of 500 random variables that are lognormally distributed about a mean and standard deviations. In this study, we assumed that each damage mode had a lognormal distribution with damage. For a given group, the percentile of the damage determines the probability of failure. The simulation shows that group #2 is more susceptible to failure while group #4 the least. A MATLAB code was written to compute the probability of failure.

## Results

The results were summarized in (Table 2) showing the age, bone mineral density (BMD), initial stiffness, final stiffness, load to failure at torsion and bending for different genders for all the four groups. BMD was measured using dual energy X-ray absorptiometry, for each bone and values entered in Table 2. It can be seen that the density for male were 1.104 ± 0.37 and 0.87 ± 0.19 for female.

**TABLE 2:** Summary of demographics and biomechanical results of the four groups.

Variable	Type of CM nail group			
	Long stainless steel Group #1	Short stainless steel Group #2	Long titanium Group #3	Short titanium Group #4
	(*n* = 14)	(*n* = 14)	(*n* = 14)	(*n* = 14)
	Mean ± SD	Mean ± SD	Mean ± SD	Mean ± SD
**Age, y**	73.5712.86	77.8613.30	75.868.25	74.2911.07
*Male*	72.5013.60	69.5013.50	75.868.25	74.5010.50
*Female*	80.000.00	81.2011.65	−	74.2011.29
**BMD, g/cm^2^**				
*Male*	1.090.43	1.360.58	0.9950.25	0.970.23
*Female*	0.800.00	0.830.25	−	0.970.32
**Initial stiffness, N/mm**	9731.392867.89	6794.272671.34	8217.514520.05	12535.976124.35
*Male*	10310.872691.65	10371.00146.00	8217.514520.05	9108.504773.50
*Female*	6254.500.00	5363.581678.66	−	13906.966067.74
**Final stiffness, N/mm**	675.75225.22	629.84262.64	655.33343.37	699.77291.25
*Male*	705.58230.11	930.46204.24	655.33343.37	490.8966.86
*Female*	496.800.00	509.60171.12	−	783.33304.20
**Load to failure**				
**At torsion, Nm**	56.1124.11	56.2523.53	76.2920.23	40.7415.05
*Male*	59.3524.60	73.6424.98	76.2920.23	39.5810.56
*Female*	36.700.00	49.2918.87	−	41.2016.48
**At bending, N**	8823.472389.71	5071.173240.29	10095.441825.06	6245.912273.03
*Male*	8480.232416.15	9594.501644.50	10095.441825.06	8686.352363.65
*Female*	10882.900.00	3261.841469.57	−	5269.741289.72
**Cadaveric femurs with no device, load to failure, N (Grote et al., 2013)**				
*Male*	4866 ± 1447.6			
*Female*	2991 ± 1045			

*n, number of samples; SD, standard deviation; BMD, bone mineral density.*

### Torsional Test

**FIGURE 3:**
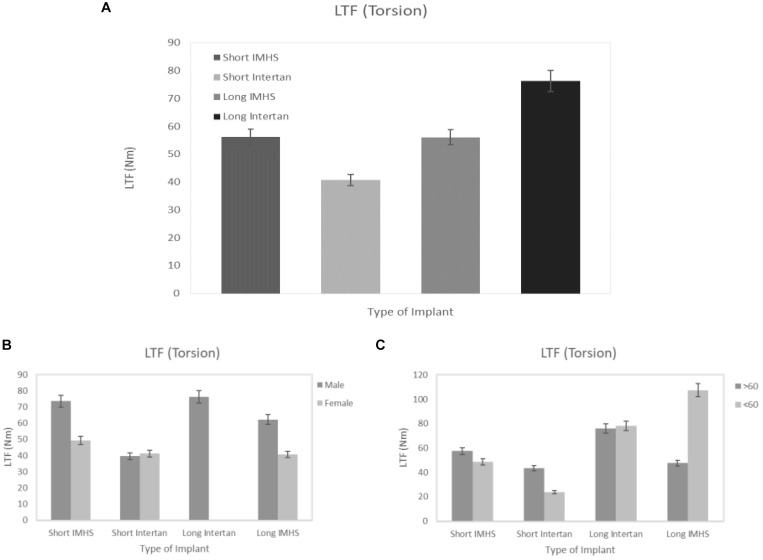
**(A)** Load to failure (LTF) in the four groups of CM nail fixation in torsion, **(B)** load to failure versus gender, and **(C)** load to failure versus two age groups.

**FIGURE 4:**
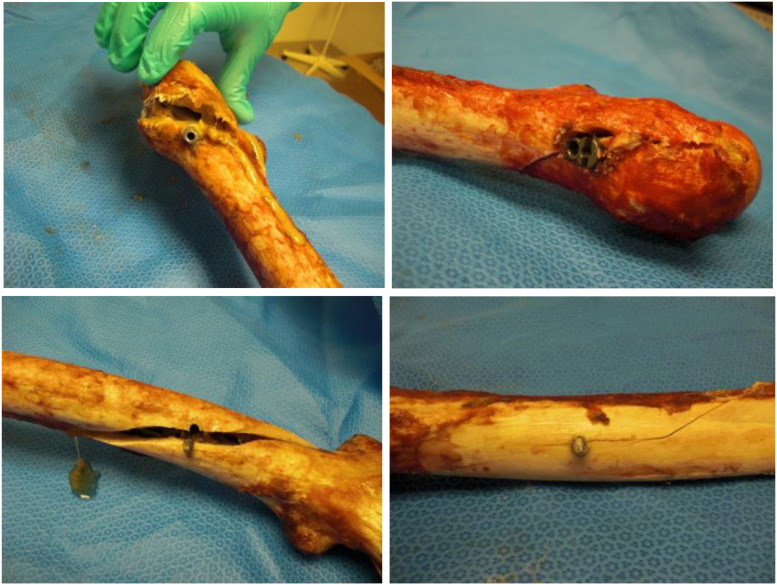
Failure at the distal screw due to torsion.

The mean torsional load to failure of long titanium and stainless steel nail groups were 76.29 ± 20.23 and 56.11 ± 24.11 Nm, respectively. In the short nails, the mean load to failure of titanium group and stainless steel group were 40.74 ± 15.05 and 56.25 ± 23.53 Nm, respectively. In general, the long titanium group had a significantly higher average load-to-failure than the short titanium (*p* < 0.05). Additionally, there was no significant difference in average load-to-failure between long titanium group and stainless-steel groups (*p* > 0.05) regardless of the length, as shown in Figure 3. These failures occurred at the screw insertion sites, as shown in Figure 4.

### Bending Test

**FIGURE 5:**
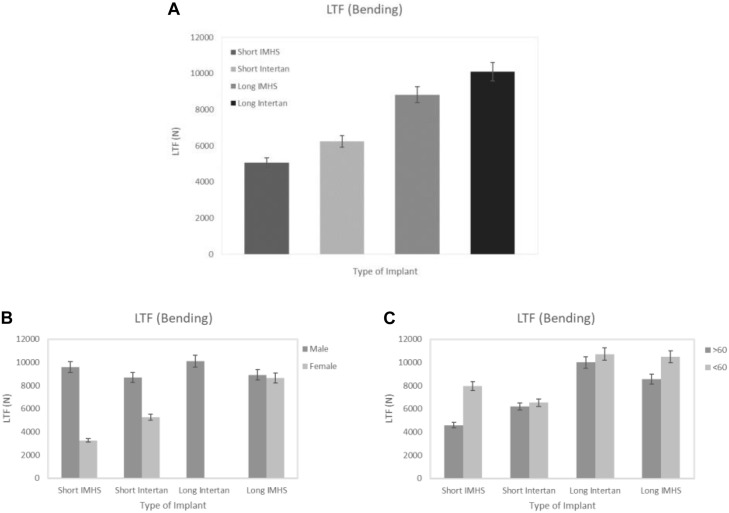
**(A)** Load to failure in the four groups of CM nail fixation in bending, **(B)** load to failure versus gender, and **(C)** load to failure versus two age groups.

**FIGURE 6:**
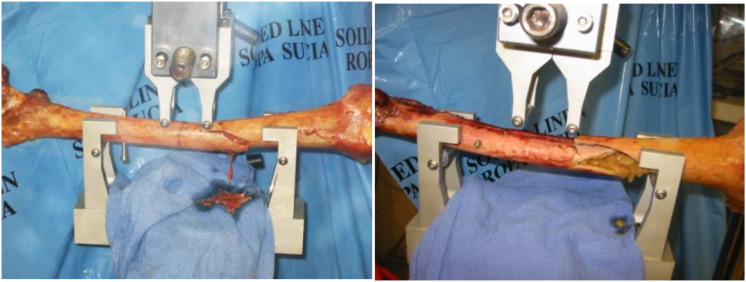
Failure along the shaft due to bending.

Similar results were found when the matched pair was tested in four-point bending. The mean flexural force to failure of long titanium and stainless-steel groups were 10,095.44 ± 1,825.06 and 8,823.47 ± 2,389.71 N, respectively. In the short nails, the mean flexural force to failure of titanium group and stainless-steel group were 6,245.91 ± 2,273.03 and 5,071.17 ± 3,240.29 N, respectively. In general, the flexural force to failure using long titanium and stainless-steel nails were approximately 160% higher than the short nails. Furthermore, the bending strength of long titanium nails were significantly higher than the short titanium nails (6,246 Nm, *p* < 0.05). Long stainless-steel nails did not differ substantially from the short titanium nails when loaded to failure in bending (*p* > 0.05) in 4-point bending tests per ASTM protocols. Length of the nail and gender had significant effects on the average force to failure in bending for all the four groups (*p* = 0.0008 and *p* < 0.001, respectively), as shown in Figure 5. Failures in four-point bending constructs occurred along the shaft distal end of the nail, oblique, however, cracks running perpendicular to the loading direction as shown in figures (Figure 6).

**FIGURE 7:**
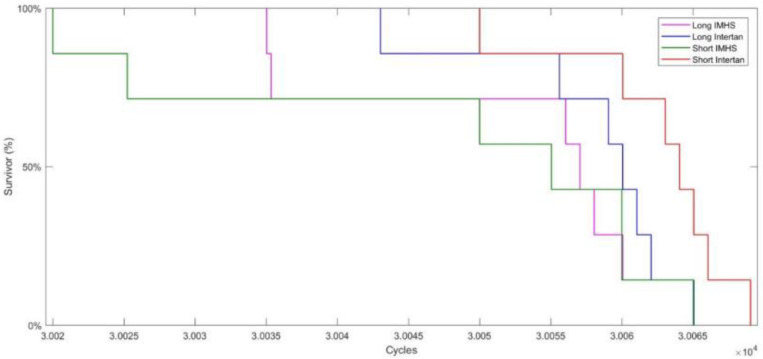
Kaplan–Meier survival analysis indicating no significant difference between the four CM fixation groups and 20% of the fixation with the four groups was estimated to survive 30,065 cycles.

Kaplan–Meier survival analysis, Figure 7, shows the differences between the number of cycles to failure in the four groups. There is no significant difference between the four CM fixation groups. Additionally, 20% of the fixation with the four groups was estimated to survive 30,065 cycles. Only 10% of the fixation with short titanium nails survived 30,070 cycles while all other types of fixation, failed below that, as shown in Figure 7.

### Damage Accumulation

#### Using Michaelis–Menten Model

**FIGURE 8:**
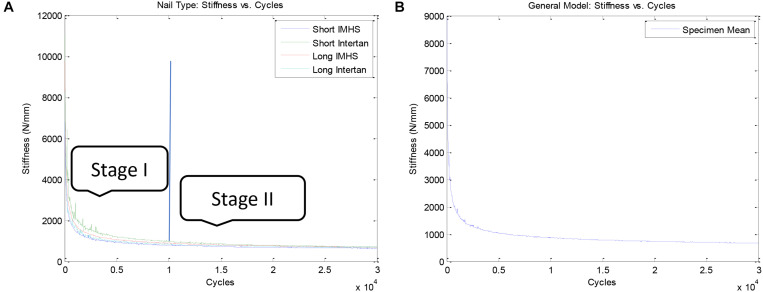
**(A)** Comparison of stiffness reduction between nail types during cyclic loading and **(B)** General model of stiffness reduction during cyclic loading.

During the initial load bear, the construct undergoes stiffness changes, where early cyclic action results in higher stiffness drop. When the stiffness behavior is modeled with respect to fatigue cycles; we observe the following behavior:

Stage I: upon execution of cyclic fatigue, majority of the stiffness reduces, observed within the first 1,000 cycles (Figure 8). Forces applied did not produce a significant change in torsional stiffness. Figure 8 shows this stiffness reduction during torsional cyclic load with constant axial load for each of the four implant types. Stiffness reduction for all implant groups was very similar supporting that the change in stiffness is due to the material properties of the bone and not due to the effect of the implant. The general trend of stiffness reduction for all of the constructs is shown in Figure 8.

Stage II: once stage I concluded, the stiffness drop with respect to number of cycles achieves a plateau and shows a steady state behavior from 1000 cycles to the end of the cyclic life, 30,000 cycles, Figure 8. This behavior can be represented by a power law equation. Therefore, the two stage stiffness drop, presents a potential parameter in which to represent damage, that accrues at different rates. Together this behavior can be modeled via Michaelis and Menten model, used in this paper.

**FIGURE 9:**
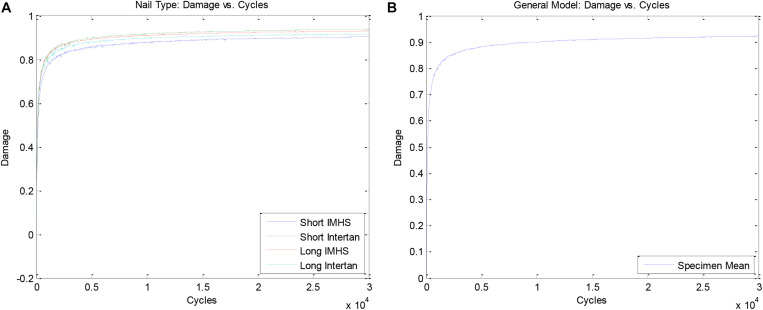
**(A)** Comparison of damage accumulation between nail types during cyclic loading and **(B)** General model of damage accumulation during cyclic loading.

Figure 9 shows damage accumulation during bi- axial, and flexural cyclic loading for each of the four implant groups. The damage accumulation for all four construct groups showing damage accumulation with respect to number of cycles is plotted in Figure 9.

**FIGURE 10:**
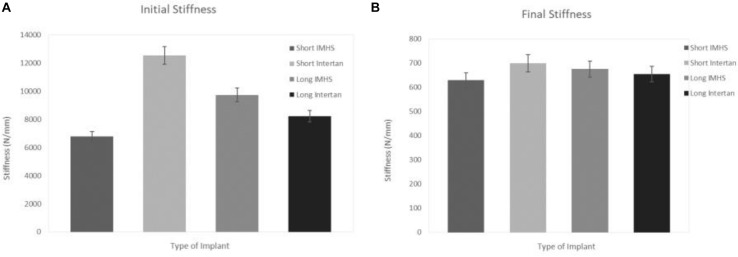
**(A)** Initial stiffness and **(B)** final stiffness in the four groups of CM nail fixation.

The mean instantaneous stiffness of long titanium and stainless-steel groups were 8,217.51 ± 4,520.05 and 9,731.39 ± 2867.89 N/mm, respectively. In the short nails, the mean initial instantaneous stiffness of titanium and stainless steel groups were 12,535.97 ± 6,124.35 and 6,794.27 ± 2671.34 N/mm, respectively. On the other hand, the average total stiffness of long titanium and stainless steel groups were 655.33 ± 343.37 and 675.75 ± 225.22 N/mm, respectively. In the short nails, the mean total stiffness of titanium group and stainless steel group were 699.77 ± 291.25 and 629.84 ± 262.64 N/mm, respectively. In general, the short titanium CM constructs had a significantly higher average instantaneous stiffness than the short stainless steel nails (*p* < 0.05). Furthermore, long titanium nails did not differ significantly from the long and short stainless-steel nails (*p* > 0.05), as shown in Figure 10.

**FIGURE 11:**
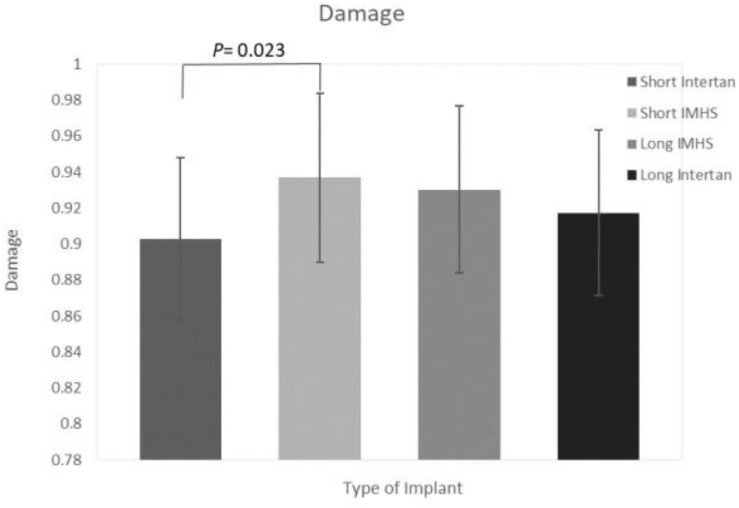
Damage in the four groups of CM nail fixation.

The damage (calculated by applying equation 1) at the end of 30,000 cycles, of long titanium and stainless steel groups were 0.92 ± 0.02 and 0.93 ± 0.01, respectively. In the short nails, the mean damage of stainless steel group and titanium group were 0.94 ± 0.02 and 0.90 ± 0.04, respectively. In general, the short stainless steel had a significantly higher average damage (0.94) than the short titanium nails (0.90, *p* < 0.05). Furthermore, long titanium nail group did not differ substantially from the short stainless steel nails (*p* > 0.05), as shown in Figure 11.

**FIGURE 12:**
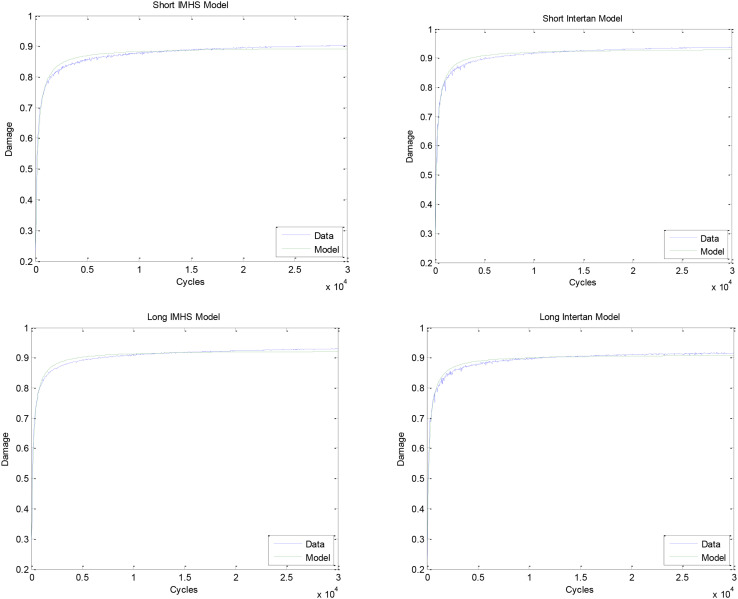
Fitted model of short IMHS nail data. Fitted model of short Intertan nail data. Fitted model of long IMHS nail data. Fitted model of long Intertan nail data. Damage was calculated using equation 1.

The damage accumulates by Stage I and Stage II process mechanisms. These two stages were modeled by non-linear regression, Michaelis–Menten model (equation 2). Two values are found from this model, including D*_*max*_* and K*_*m*_*. The maximum damage that is approached and the number of cycles at half the maximum damage is listed in Table 3. The graphs of these models for each of the nail types and the composite model are shown in Figure 12.

**TABLE 3:** D_*max*_ and K_*m*_ for each of the nail types and the general model.

Model	D_*max*_ (95% CI)	K_*m*_ (95% CI) cycles
Short IMHS Model	0.90 (0.90, 0.90)	157.74 (154.74, 160.74)
Short Intertan Model	0.93 (0.93, 0.93)	130.97 (128.51, 133.43)
Long IMHS Model	0.92 (0.92, 0.93)	126.86 (124.75, 128.98)
Long Intertan Model	0.91 (0.91, 0.91)	129.82 (127.64, 131.99)
Composite Model	0.92 (0.92, 0.92)	135.82 (133.50, 138.14)

**FIGURE 13:**
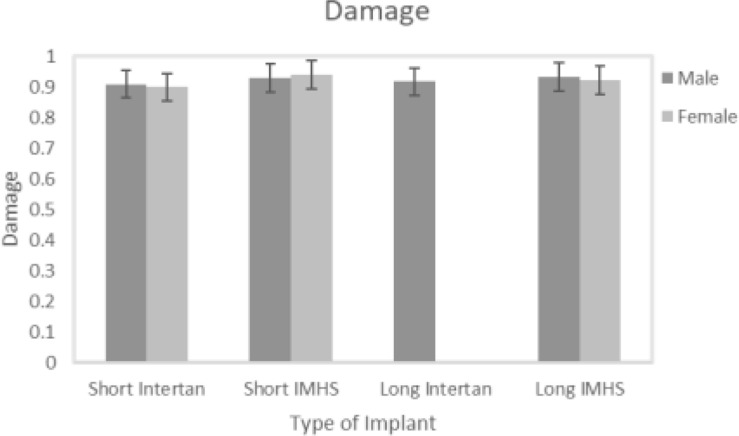
Damage versus gender.

Short stainless steel nail group showed the highest mean damage accumulation compared to the other three groups. On the other hand, short titanium group showed the least mean damage accumulation. Despite the non-significant difference in damage accumulation with respect to genders in the four groups (*p* = 0.64), results showed gender had a significant effect on load to failure in both bi-axial and 4-point bending tests (*p* = 0.025 and *p* < 0.001, respectively), as shown in Figure 13.

**FIGURE 14:**
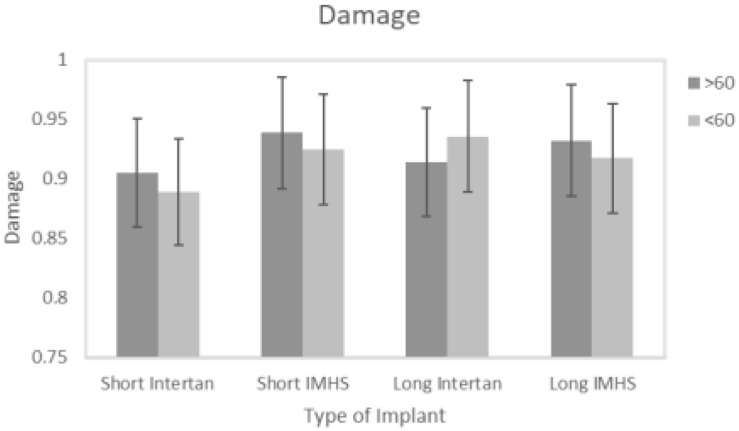
Damage versus age.

Even though no significant difference was observed in damage accumulation results when age was the factor, there are two points that need to be considered. All the groups showed high load to failure in both torsional and bending tests and eventually higher damage accumulation when patients ages were greater than 60 years except for group #3. Secondly, this group also showed higher load to failure and damage accumulation with lower than 60 years age with higher BMD, that makes it more susceptible to failure compared to other fixation groups, as shown in Figure 14.

**FIGURE 15:**
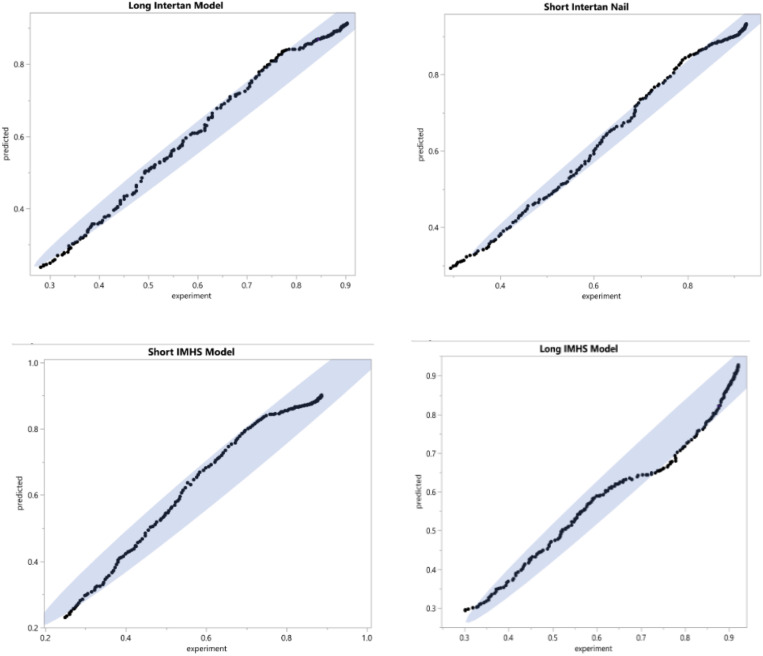
The difference between the experimental data and the predicted data for each type of internal fixation nail constructs (95% confidence interval).

**FIGURE 16:**
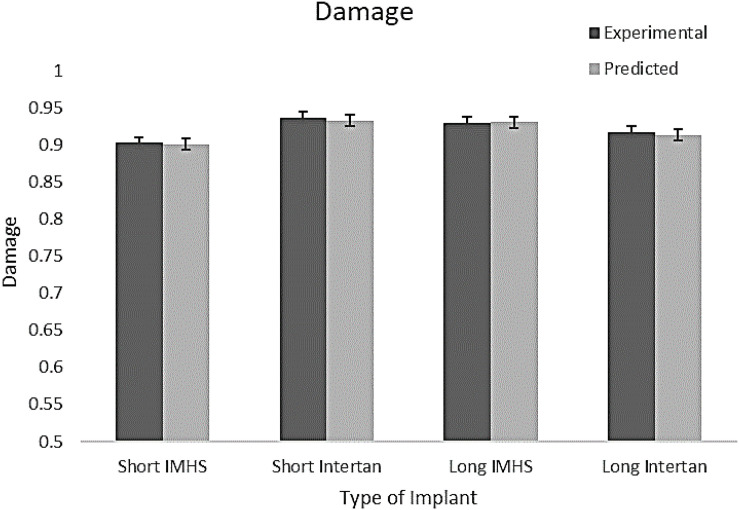
Experiential and predicted damage in the four groups of CM nail fixation.

The applicability of the prediction model to represent experimental data was determined statistically. The analysis demonstrated that one model can predict the damage accumulation during cyclic loading for the experimental nailing fixation for all four groups. Figure 15 shows the predicted versus experimentally obtained damage in short and long nail groups. The residuals are within the 95% confidence interval, thus can be used in the design of such implant systems. Therefore, the prediction tool used to model the damage development is applicable to all the four groups tested experimentally with the long and the short nails. In addition, Table 4 shows the statistical results for each type of fixation. For the four groups, the damage found experimentally was compared with predicted data, as shown in Figure 16.

**TABLE 4:** The statistical results for each type of fixation showing that there is no significant difference between the experimental and predicted damage models.

Fixation	*F*-test	*P*-value	R square
Short IMHS	0.08	0.78	0.0001
Short Intertan	0.07	0.76	0.0005
Long IMHS	0.10	0.81	0.0003
Long Intertan	0.06	0.79	0.0003

#### Damage Accumulation – Phenomenological Model

A Regression model was developed for bone damage accumulation with respect to axial stiffness and bone mineral density using MATLAB, where the regression model is:

(5) $$D=0.93-\left(0.01\,\,\xi_{A}\right)+\left(0.03\,\rho \right)-\left(0.003\,\,\xi_{A}^{2}\right)+\left(0.023\,\xi_{A}\,\rho \right)-\left(0.013\,\rho^{2}\right)+\left(0.008\,\,\xi_{A}^{3}\right)-\left(0.013\,\xi_{A}^{2}\,\rho \right)-\left(0.0055\,\,\xi_{A}\,\rho^{2}\right)$$

**FIGURE 17:**
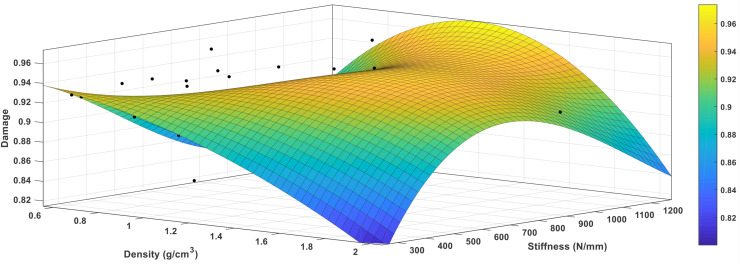
The sensitivity plot demonstrating the relation between damage, density, and stiffness.

Where SSE: 0.015, R-square: 0.81, Adjusted R-square: 0.80, and RMSE: 8.03. This model can be used to predict the damage accumulation of the bone with respect to different bone densities. Figure 17 illustrates the sensitivity plot demonstrating the relation between stress, strain, and orientation angle.

**FIGURE 18:**
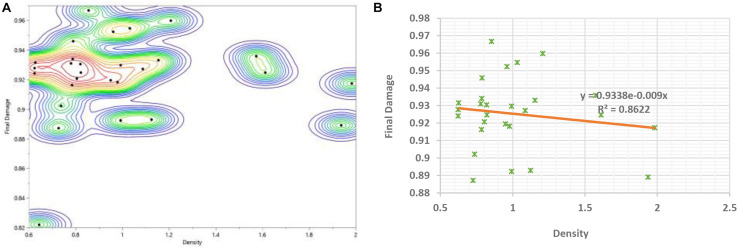
**(A)** Bivariate fit of final damage by density **(B)** Final damage versus density showing the exponential fit with the analytical equation.

A comparison between the prediction profiler of damage accumulation with respect to load to failure in both bending and torsion was performed and found to be a function of stiffness parameters and density. Figure 18 shows the Bivariate Fit of damage by density, and illustrates that damage increases as the density decreases. The bivariate fit of damage by density was represented for each type of nail fixation and for both genders (see Appendix I). The prediction expression of damage accumulation with respect to load to failure in bending is as follows:

(6) $$D=0.910+7.49\times 10^{-6}\xi_{A\,O}+0.00012\xi_{A}+2.39\times 10^{-9}\times \left({\text{ξ}}_{A\,O}-9342.54\right)\times \left({\text{ξ}}_{A}-666.53\right)+5.22\times 10^{-7}\times {ℒ}_{B}+5.87\times 10^{-9}\times \left({\text{ξ}}_{A}-666.53\right)\times \left({ℒ}_{B}-7575.03\right)+0.013\rho +4.97\times 10^{-6}\times {\left({ℒ}_{B}-7575.03\right)}^{*}\,\,\left(\rho -1.0023\right)$$

Additionally, the prediction expression of damage accumulation with respect to load to failure in torsion is as follows:

(7) $$D=0.93+6.69\times 10^{-6}\xi_{A\,O}+0.0001{\text{ξ}}_{A}+0.0002{ℒ}_{T}+6.85\times 10^{-8}{\left({\text{ξ}}_{A\,O}-9342.54\right)}^{*}({ℒ}_{T}-57.58+1.046\times 10^{-6}{\left({\text{ξ}}_{A\,O}-666.53\right)}^{*}\,\,\left({ℒ}_{T}-57.58\right)+0.0078\,\rho +0.00035{\left({ℒ}_{T}-57.58\right)}^{*}\,\,\left(\rho -1.0023\right)$$

#### Damage Accumulation – Using Probabilistic Monte Carlo Simulation

**FIGURE 19:**
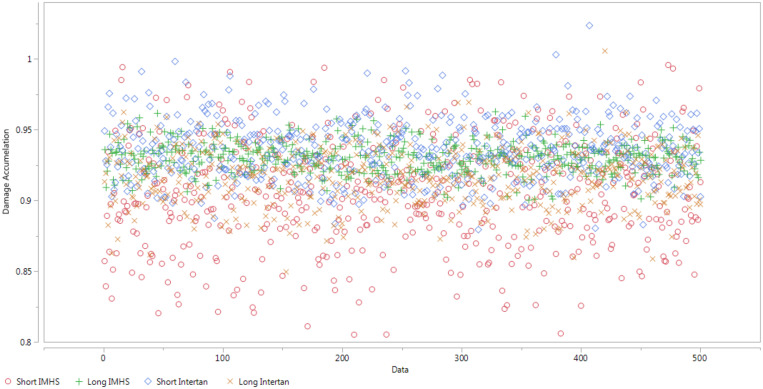
Damage accumulation for each CM nail through Monte Carlo simulations for 500 variables.

**FIGURE 20:**
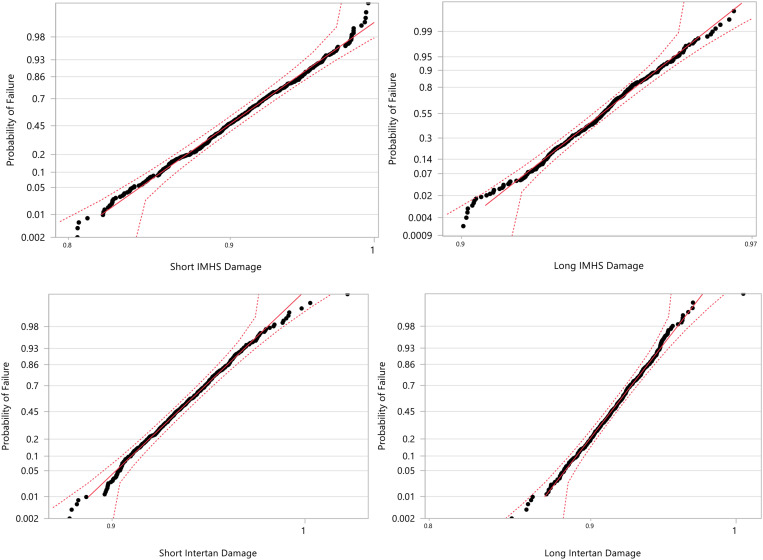
A lognormal distribution curves for each CM nail damage through Monte Carlo simulations for 500 variables.

The Monte Carlo simulations were performed to produce a set of 500 random variables that are lognormally distributed about a mean and standard deviations (shown in Figure 19). The output data for each group type was plotted in terms of actual values versus the probability of failure using JMP^®^14 software (Figure 20). Based upon this analysis we observe that higher probability of failure in experimental data occurs at achieving 50–60% of the life (15,000–18,000 cycles). The damage at this life is 0.5 with 70% probability that failure may occur. This should be used as a criterion in preparing the constructs with the given nailing systems. Additionally, the parameter estimates for each CM nail type are shown in Table 5. Finally, the probability of failure was compared with age for both genders. The comparison illustrates that the construct probability of failure increases for the age group 70–90 years old for both genders.

**TABLE 5:** Parameter estimates (lognormal distribution) for each CM nail type.

Nail type	Type	Parameter	Estimate	Lower 95%	Upper 95%
Short IMHS	Scale	μ	–0.10	–0.11	–0.10
	Shape	σ	0.04	0.04	0.04
Long IMHS	Scale	μ	–0.07	–0.07	–0.07
	Shape	σ	0.01	0.01	0.01
Short Intertan	Scale	μ	–0.07	–0.07	–0.06
	Shape	σ	0.02	0.02	0.02
Long Intertan	Scale	μ	–0.09	–0.09	–0.09
	Shape	σ	0.02	0.02	0.02

## Discussion

To the best of our knowledge, this study is the first that evaluates the peri- implant/prosthetic failure of in-tact femora constructed with short and long CM nails. Implant length, material of construction with Ti-6AL-4V and SS-316L, bone quality determined by age, and gender parameters were included in the testing protocol under static (flexural force to failure and axial force to failure) of pre-fatigued constructs under bi-axial and bending conditions. These conditions simulated cyclic axial and torsion loading, and femoral bending as a result of load bear. The reduction of fracture-gap was proposed to be a function of construct cyclic, instantaneous stiffness. This study was designed to investigate the effect of dynamic stiffness under cyclic loading and that stiffness reduces abruptly with the application of cycles to about 1000 cycles, then stabilizes and achieves a plateau. An empirical equation (1) proposed to describe damage development, which was assumed to be 0 at the beginning of the cycling and reaching a critical value at the end of 30,000 cycles. This behavior was modeled using Michaelis–Menten modeling, phenomenological modeling that uses critical test parameters, and probabilistic Monte Carlo simulation.

Literature shows that clinical advantages of the constructs made with short nails with their counterparts, namely long nails; present shorter operation time that is beneficial for patients with multiple trauma, special anesthesia requirements, or patients with severe medical conditions. Literature indicated that long and short CM nails had similar benefits for older patients (>65) despite the biomechanical differences (Hou et al., 2013; Boone et al., 2014; Kleweno et al., 2014). We found significant differences between short titanium group #4 and other CM groups #1–3. Additionally, we found that gender had a significant effect on damage accumulation in groups 2 and 4, respectively.

It can be seen from Table 2 that the density for male femurs were 1.104 ± 0.37 and 0.87 ± 0.19 for female. Group #3 showed higher load to failure in both bending and torsion and damage accumulation with lower than 60 years age with higher BMD, that makes it more susceptible to failure compared to other fixation groups. We assumed that titanium and stainless steel nails are biomechanically different and age and gender could offer significant effects on the damage accumulation. Nail design does not have significant effects clinically (Marmor et al., 2015). However, their work was based on synthetic bones. Performing the study on cadaver femur has its advantages in observing the demographical factors and their effects on damage accumulation. It is noteworthy that, despite some studies focused on biomechanical characterization of CM nail for femoral fractures, limited information is available on cyclic fatigue damage accumulation in femoral bone as a result of partial/full-load bear.

We found that group #3 had significantly higher load-to-failure than the group #4 when loaded to failure in torsion. Additionally, there was no significant difference in average load-to-failure between groups #1 and 3 regardless of the length. Long stainless-steel nails (#1) did not differ substantially from the short titanium nails (#4) when loaded to failure in bending. Furthermore, length and gender had significant effects on the average load to failure in bending for all the four groups. Analysis of the stiffness shows that stage I of cyclic fatigue, where the majority of stiffness reduction occurs, is completed within the first 1,000 cycles and the forces applied did not produce a significant change in torsional stiffness. Kaplan–Meier analysis indicated that there is no significant difference between the four CM fixation groups and only 20% of the fixation with the four groups is estimated to survive after 30,065 cycles. In general, bone with no device had x-2.64 lower load to failure in both bending and torsion than the bone with long nail constructs and x-1.79 lower than the bone with short nail constructs.

**FIGURE 21:**
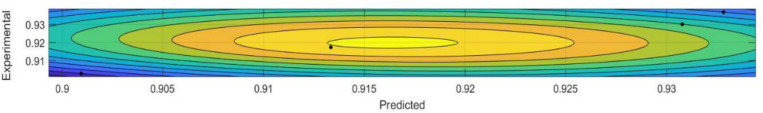
Contour plot of experiential versus predicted damage in the four groups of CM nail fixation.

Group #2 indicated the highest mean damage accumulation compared to the other three groups. On the other hand, Group #4 showed the least mean damage accumulation using the three frameworks in which damage accumulation was modeled. Despite the non-significant differences in damage accumulation with respect to gender in the four groups, results showed gender had a significant effect on load to failure in both torsional and bending tests. Even though no significant difference was observed in damage accumulation results when age was the factor, Group #3 showed higher load to failure in both bending and torsion and damage accumulation with ages younger than 60 years were more susceptible to failure compared to other fixation groups. Michaelis–Menten model was found to be applicable to all four groups of testing, where the composite model developed here can predict the damage development in femoral constructs and delay load bear if needed. Figure 12 showed that damage accumulation occurs at high rates from the start of cyclic activity. These design charts will be very useful in the design of devices and in the pre-planning of the surgery allowing rehab activities. Figure 21 shows the contour of the sensitivity plot showing that composite model is able to predict damage in all four groups.

While this study is the first study that observes damage accumulation of different fixation devices using cadaver bone samples and considering age and gender factors, some limitations were encountered. The male and female bone samples have not been distributed evenly between the four groups because the samples were randomized to receive fixation. Because of that, long titanium had only male sample and was excluded from the evaluation of gender factor on damage accumulation. Another limitation of our study is due to performing the experiments in non-fractured state. As our investigation focused on evaluating CM nail constructs with regard to post-primary healing to determine the risk of peri-prosthetic fractures, no fracture was introduced to the cadaveric femora. Because of that, the effect of fracture type and the severity of bone failure on damage accumulation were not included in our investigation, as our study focuses on the device performance rather than fracture treatment.

The Monte Carlo simulations produced a set of 500 random variables that are lognormally distributed about a mean and standard deviations. The simulation shows that short titanium CM nail is the least susceptible to failure and short stainless steel is the most susceptible to failure. The probability of failure was compared with age for both genders. The comparison illustrates that the device probability of failure increased for the age group 70–90 years old for both genders with higher probability to failure.

Our study illustrates the efficacy of short titanium CM nails, group #4, compared to short stainless steel, long titanium, long stainless steel nails for femoral fixation and the effect of age and gender on damage accumulation. We recommend that clinical decision should take age and gender into consideration before the implant selection is made.

## Conclusion

1. *In vitro* biomechanical models of the CM nail constructs that were investigated with regard to post-primary healing to determine the risk of peri-prosthetic fractures offer insights in understanding the underlying mechanisms of injury and dysfunction, leading to improved prevention, diagnosis, and treatment of bone problems.
2. Analysis of the stiffness shows that stage I of cyclic fatigue, where the majority of stiffness reduction occurs, is completed within the first 1,000 cycles, and forces applied did not produce a significant change in torsional stiffness.
3. Our investigation supports the use of short titanium CM nail, as Kaplan–Meier survival analysis illustrated that this nail type is the least susceptible to failure for the fixation of femoral fractures when the use of long or short nails is possible.
4. Our analysis demonstrated that the clinical decision should take age and gender into consideration before the implant selection is made.
5. Mathematical models and regression equations would be beneficial in developing a novel procedure to predict the failure of the bone/device constructs to make clinical decisions in the operating room.

## Data Availability Statement

The original contributions presented in the study are included in the article/supplementary material, further inquiries can be directed to the corresponding author.

## Ethics Statement

The studies involving human participants were reviewed and approved by the Clinical Ethical Committee of the Miami Valley Hospital. The cadaver material was procured from Anatomical Gift Program of Wright State University, Dayton, OH, United States to the Orthopaedic Surgery, Sports Medicine and Rehabilitation Department for the investigation. Written informed consent for participation was not required for this study in accordance with the national legislation and the institutional requirements.

## Author Contributions

MP designed the clinical aspects of the research, obtained the devices from Smith and Nephew, and supervised resident research (MS) who prepared the device constructs. TG led the biomechanical evaluation of the project and necessary fatigue interpretations along with advising resident, as well as Ph.D. student AW who performed the tests. FH, Ph.D. student of TG, reduced the data, performed all the modeling and simulations, and prepared the manuscript with TG. JR provided necessary data on the *in vivo* behavior and gait. All authors contributed to the article and approved the submitted version.

## Conflict of Interest

The authors declare that the research was conducted in the absence of any commercial or financial relationships that could be construed as a potential conflict of interest.
